# Post-transplant laronidase augmentation for children with Hurler syndrome: biochemical outcomes

**DOI:** 10.1038/s41598-019-50595-1

**Published:** 2019-10-01

**Authors:** Troy C. Lund, Weston P. Miller, Ai Yin Liao, Jakub Tolar, Ryan Shanley, Marzia Pasquali, Nicole Sando, Brian W. Bigger, Lynda E. Polgreen, Paul J. Orchard

**Affiliations:** 1 University of Minnesota, Division of Pediatric Blood and Marrow Transplant, Minneapolis, 55455 USA; 2 University of Minnesota, Division of Biostatistics, Minneapolis, 55455 USA; 3 University of Manchester, Manchester, M13 9PL United Kingdom; 4 University of Utah, Salt Lake City, 84112 USA; 5 Los Angeles Biomedical Research Institute at Harbor-UCLA Medical Center, Torrance, 90502 USA; 6 Sangamo Therapeutics, Richmond, 94804 USA

**Keywords:** Genetics, Medical genetics

## Abstract

Allogeneic hematopoietic cell transplantation (HCT) benefits children with Hurler syndrome (MPS-IH). However, survivors remain burdened by substantial MPS-IH related residual disease. We studied the feasibility, safety and biochemical impact of augmentative recombinant intravenous enzyme replacement therapy (IV-ERT) post transplantation. Ten children with MPS-IH and ≥2 years from successful HCT underwent IV-ERT for 2 years’ duration. Patients were monitored for anti-drug antibody (ADA) development, including inhibitory capacity and changes in urinary excretion of glycosaminoglycans (uGAG). Three patients demonstrated low-level ADA at baseline, though all children tolerated IV-ERT well. Eight patients developed ADA over the 2-year study, with 3 (38%) meeting criteria for an inhibitory ADA response. The aggregate cohort experienced a reduction in uGAG from baseline to study end, which was enhanced in children with low or no ADA response. Conversely, children with inhibitory ADA showed increase in uGAG over time. IV-ERT in previously transplanted children with MPS-IH appears safe and can reduce uGAG, although this is reversed by the presence of inhibitory ADA. These data show a biochemical change after initiation of post-HCT IV-ERT, but the occurrence of ADA and inhibitory antibodies are a concern and should be monitored in future efficacy trials. This trial was registered at www.clinicaltrials.gov, NCT01173016, 07/30/2010.

## Introduction

Severe mucopolysaccharidosis type I, or Hurler syndrome (MPS-IH), is a rare, autosomal recessive disorder of glycosaminoglycan (GAG) catabolism. Affected patients demonstrate low-to-absent functional α-L-iduronidase activity with resulting pathologic accumulation of GAG. If untreated, the disease is characterized by progressive and severe organ dysfunction (cardiac, airway/pulmonary, central nervous system [CNS], musculoskeletal) and typically results in death within the first decade^1^. Since the first report of the use of allogeneic hematopoietic cell transplantation (HCT) for MPS-IH in 1981, HCT has emerged as the recommended therapy for children diagnosed at an early age and yet to experience significant neurodevelopmental decline^2^. HCT attenuates disease as donor-derived hematopoietic cells serve as a source of functional α-L-iduronidase for the degradation of GAG within recipient somatic and CNS tissue^3^. Over the past 3 decades, engrafted survival outcomes have improved following HCT for children with MPS-IH^4,5^. However, as these patients live longer, it has become evident that transplant does not fully ameliorate the disorder. In a recent, retrospective, multi-center study of 217 patients with MPS-IH surviving HCT, investigators catalogued high incidences of substantial and morbid residual Hurler-related disease. Importantly, they correlated increased disease burden with lower post-transplant circulating leukocyte α-L-iduronidase activity, a finding begging the clinical question of whether augmentative enzyme sources might be beneficial^6^.

Laronidase is a recombinant α-L-iduronidase product licensed for intravenous enzyme replacement therapy (IV-ERT) in all phenotypes of MPS-I, including Hurler syndrome, the severest disease form. Laronidase is alone insufficient to treat the CNS disease associated with MPS-IH as it does not adequately penetrate the blood-brain barrier. However, laronidase IV-ERT has been incorporated into peri-transplant regimens by many centers on the basis that it might improve pre-transplant somatic disease and decrease transplant-related complications. Initial reports suggest this to be a safe and effective strategy, even as the immunogenic potential of IV-ERT to elicit inhibitory anti-drug antibodies (ADA) in this setting is documented^7,8^. However, for patients with MPS-IH who have undergone successful HCT in the distant past, studies of the use of laronidase IV-ERT to augment therapy are lacking. We evaluated the safety, immunogenicity and biochemical effect of IV-ERT in patients with MPS-IH who were 2 years or greater from HCT with evidence of donor engraftment. Here we describe the incidence and impact of ADA, including inhibitory capacity, on donor hematopoietic chimerism and urinary GAG excretion (uGAG) over two years’ duration of laronidase treatment.

## Results

Ten children with MPS-IH underwent post-transplant IV-ERT augmentation of and completed 2 years of ERT on this study. The median age at the initiation of ERT augmentation was 9.5 years (range, 5.1 to 13.8 years). The median time from HCT to study entry was 3.7 years (range, 2.4 to 13.1 years). While on study, one patient had a seizure thought to be unrelated to IV-ERT treatment. Table 1 shows patient- and disease-related characteristics at study entry, as well as the number of doses of drug not administered.

**Table 1:** Baseline characteristics at study entry of 10 children with Hurler syndrome receiving post-transplant IV-ERT augmentation.

ID	Prior ERT	Sex	Age at HCT	HCT Graft	Age at Study	Baseline ADA (titer)	IDUA activity	Chimerism (% donor)	Baseline Urine GAG (mg/mmol creatinine)			% Doses Missed
									HS	I0S0	I0S6	
001	no	F	8 m	URD	13y	0	nl	79%	1.30	0.03	0.18	0%
002	yes	M	33 m	UCB	5y	0	nl	99%	0.67	0.01	0.15	1%
004	yes	F	35 m	UCB	5y	400	low	77%	1.70	0.07	0.40	0%
005	yes	M	16 m	UCB	8y	100	nl	81%	0.61	0.01	0.10	11%
006	no	F	2 m	UCB	10y	0	nl	72%	0.76	0.02	0.10	6%
007	no	M	32 m	RD	13y	0	low	90%	1.30	0.04	0.23	6%
008	no	M	9 m	UCB	11y	0	nl	100%	0.61	0.01	0.11	1%
009	yes	F	24 m	RD	10y	0	low	100%	0.91	0.02	0.14	1%
010	no	M	31 m	RD	8y	100	nl	100%	0.74	0.02	0.12	0%
011	yes	M	7 m	RD	9y	0	nl	100%	1.10	0.04	0.18	0%

F = female; M = male; m = months; URD = unrelated donor; UCB = umbilical cord blood; RD = related donor; y = years at study entry; ADA = anti-drug antibody titer by commercial assay; IDUA = leukocyte iduronidase activity; nl = within normal limits; low = lower than normal range; GAG = glycosaminoglycans; HS = heparan sulfate; I0S0 and I0S6 denote MPS-I-specific non-reducing ends.

**Figure 1:**
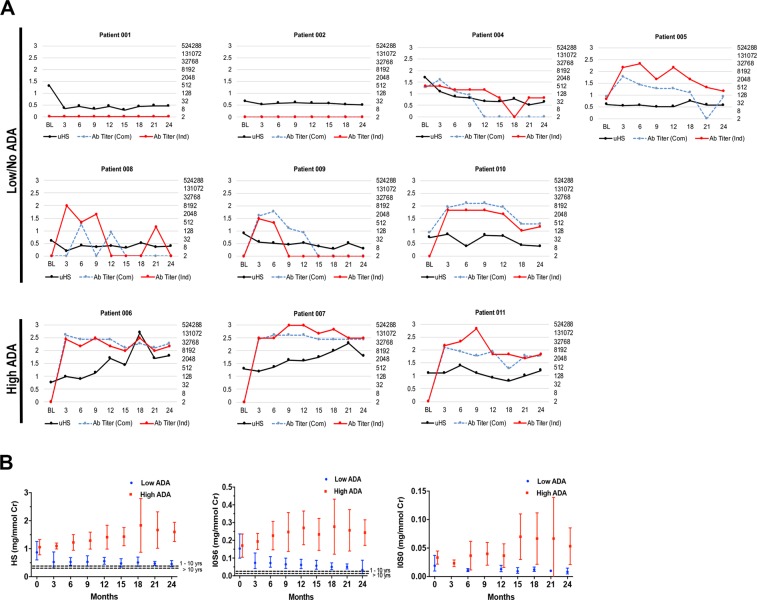
Individual patient anti-drug antibody response and urine biomarker excretion over time. (**A**) BL (baseline) indicates data acquisition just prior to the first dose of IV laronidase. Numbers on the x-axes indicate months on study. Left y-axes show urine heparan sulfate excretion in mg/mmol creatinine (black line). Right y-axes (logarithmic scale) show anti-laronidase antibody (ADA) titers, blue dashed line shows the titer of commercial (Com) assay, the red solid line shows independently performed titers (Ind). (**B**) Excretion of heparan sulfate, NRE I0S6, and NRE i0S0. Circles indicated geometric mean values; whiskers show 95% confidence intervals. Note that the geometric mean is not defined when any value is zero or negative (so is left blank). See Table 3 for statistical comparisons of baseline values to those at 24 months. The non-MPS values are shown as dashed lines in the graphs. I0S0 non-MPS is not detectable.

Three patients (ID’s 004, 005, 010) demonstrated low-titer ADA at baseline prior to beginning IV-ERT augmentation with a maximum observed titer of 400 by a commercial assay (Com) and 512 by an independent assay (Ind) (Table 1 and Fig. 1A). The remaining 7 patients had no measurable ADA at enrollment. When comparing the patients with and without baseline ADA, there were no significant differences in pre-treatment urinary excretion of heparan sulfate (HS), I0S0, or I0S6; however, patients without baseline ADA had slightly higher donor hematopoietic chimerism at enrollment (91% versus 86%; p < 0.01). Three patients (ID’s 004, 007, 009) had baseline leukocyte α-L-iduronidase activity levels below the lower limit of normal (Table 1). Two of these patients previously underwent related-donor HCT, and both donors demonstrated normal leukocyte iduronidase activity. The third patient was transplanted with unrelated umbilical cord blood unit. All individuals showed predominant donor hematopoietic engraftment of at least 75%.

Two patients (ID’s 001 and 002) mounted no anti-laronidase ADA response for the entirety of the study (neither apparent at baseline nor at subsequent testing). Of the remaining 8 patients, 3 developed high ADA responses (ID’s 006, 007, and 011). Individual patient maximal ADA titers as measured by the independent assay, along with concurrent values from the commercial assay, and ADA inhibitory function data are shown in Table 2.

**Table 2:** Determination of high ADA responders versus low/no ADA responders for 10 children with Hurler syndrome receiving post-transplant laronidase IV ERT augmentation.

ID	Maximum Anti-drug Antibody^@^			Month 24 Anti-drug Antibody			ADA Classification
	Titer (Com)	Titer (Ind)	% Inhibition	Titer (Com)	Titer (Ind)	% Inhibition	
001	0	0	—	0	0	—	Low/No
002	0	0	—	0	0	—	Low/No
004	1,600	512	—	0	64	—	Low/No
005	800	32,768	18%	100	256	0%	Low/No
006	51,200	65,536	56%	25,600	16,384	94%	High
007	102,400	524,288	87%	51,200	65,536	98%	High
008	0	8,192	10%	0	0	9%	Low/No
009	1,600	1,024	0%	0	0	0%	Low/No
010	12,800	4,096	46%	400	256	0%	Low/No
011	3,200	262144	40%	3,200	4,096	23%	High

^@^Each patient’s maximum antibody titer time-point as determined by the independent assay (Ind); the concurrent titer from the commercial assay (Com) is shown for comparison; ADA = anti-drug antibody.

Changes over time in urine HS excretion, as well as ADA titers measured by both assays, are shown for each patient in Fig. 1A. When considering the entire cohort, significant decrements in intra-patient uGAG (HS, I0S0, and I0S6) were observed between baseline and study’s end (month 24) (Table 3 and Fig. 1B). When considering only patients with a low ADA response (n = 7), this effect was enhanced. In contrast, for patients with a high ADA response (n = 3), significant intra-patient increase in urinary HS excretion was observed between baseline and study’s end. For this same group, urinary excretion of I0S0 and I0S6 appeared higher at month 24 than baseline, though the intra-patient difference did not reach statistical significance.

**Table 3:** Urinary excretion of glycosaminoglycan biomarkers at baseline and after 2 years of IV-ERT augmentation using a generalized estimated equations model to assess intra-patient changes.

Condition	n	Baseline GM (95% CI)	Month 24 GM (95% CI)	GM Ratio (95% CI)	p-value (ratio)
**Heparan sulfate (mg/mmol creatinine)**					
All	10	0.91 (0.74, 1.12)	0.66 (0.57, 0.76)	0.72 (0.58, 0.90)	<0.01
Low/No ADA	7	0.87 (0.66, 1.14)	0.45 (0.38, 0.54)	0.52 (0.39, 0.69)	<0.01
High ADA	3	1.03 (0.80, 1.33)	1.57 (1.27, 1.95)	1.53 (1.06, 2.21)	0.02
**I0S6 (mg/mmol creatinine)**					
All	10	0.16 (0.12, 0.20)	0.07 (0.06, 0.09)	0.48 (0.38, 0.60)	<0.01
Low/No ADA	7	0.15 (0.11, 0.21)	0.05 (0.04, 0.06)	0.29 (0.24, 0.37)	<0.01
High ADA	3	0.16 (0.11, 0.24)	0.24 (0.17, 0.32)	1.46 (0.83, 2.58)	0.19
**I0S0 (mg/mmol creatinine)**					
All	10	0.022 (0.015, 0.032)	0.014 (0.010, 0.020)	0.627 (0.408, 0.963)	0.03
Low/No ADA	7	0.019 (0.011, 0.031)	0.008 (0.005, 0.013)	0.431 (0.245, 0.759)	<0.01
High ADA	3	0.032 (0.022, 0.046)	0.048 (0.028, 0.081)	1.500 (0.859, 2.619)	0.15

GM = geometric means from the generalized estimated equations model; GM ratio = ratio of geometric means at 24 months compared to baseline; CI = confidence interval; ADA = anti-drug drug antibody; p-values are from tests of the null hypothesis that the true GM ratio (month 24/baseline) is 1.

No significant changes in donor hematopoietic chimerism were noted between study enrollment and study’s end (month 24) for the entire cohort, and this observation was independent of ADA or inhibitory antibody status (Table 4).

**Table 4:** Donor hematopoietic chimerism at baseline and after 2 years of IV-ERT augmentation using a generalized estimated equations model to assess intra-patient changes.

Condition	n	Baseline GM (95% CI)	Month 24 GM (95% CI)	GM Ratio (95% CI)	p-value (ratio)
All	10	89 (83, 96)	86 (76, 97)	0.97 (0.91, 1.03)	0.28
Low/No ADA	7	90 (83, 98)	86 (74, 101)	0.96 (0.87, 1.05)	0.32
High ADA	3	87 (74, 101)	86 (71, 103)	0.99 (0.95, 1.03)	0.54

GM = geometric means from the generalized estimated equations model; GM ratio = ratio of geometric means at 24 months compared to baseline; CI = confidence interval; ADA = anti-laronidase drug antibody; p-values are from tests of the null hypothesis that the true GM ratio (month 24/baseline) is 1.

## Discussion

Based on clinical studies documenting the benefit of full engraftment and higher enzyme levels positively influencing long term outcomes^6^, and animal data suggesting a dose-response effect of enzyme delivery^9^, we hypothesized that there may be better biochemical correction with the addition of IV-ERT after transplant. Our study is the first to test the biochemical changes that accompany the addition of IV-ERT after allogeneic transplantation for Hurler syndrome. We show that eight patients mounted anti-laronidase antibody responses while on study; only 3 of these patients met high inhibitory ADA response criteria as previously reported^10^. Intra-patient reductions in uGAG between baseline and study’s end (month 24) were observed for the aggregate cohort. This effect was strengthened among 7 patients in whom no or low-ADA response occurred. Importantly, however, in the 3 patients with high inhibitory ADA response, the effect was not only negated, but an aggregate increase in uGAG (from baseline to month 24) was observed (Fig. 1; Table 3). For the collective cohort, independent of ADA response, the donor hematopoietic graft remained stable and appeared unaffected by IV-ERT augmentation.

Urinary GAG excretion is a well-described biomarker in both untreated and treated MPS-IH, and it is known that patients demonstrate uGAG reduction, along with clinical benefit, following either IV-ERT or HCT^11–14^. Some data suggest that HCT provides superior uGAG reduction compared to IV-ERT, while other reports have shown that HCT adds a step-wise, incremental reduction in uGAG when it follows IV-ERT^8,15^. However, few studies have correlated the magnitude of a clinical benefit with the degree of reduction in uGAG, regardless of the therapeutic intervention achieving it.

In an analysis of children undergoing sequential IV-ERT followed by HCT, Saif and colleagues demonstrated universal ADA response in the window between ERT initiation and HCT^7^. When limited to receiving ERT prior to transplantation, most children experienced ADA loss by 3 months after HCT, and all were ADA negative by 2 years following transplant. Most patients’ ADA were determined to be inhibitory, either for enzymatic catalytic function or cellular uptake^7^.

The clinical consequences of ADA in MPS-IH patients, either in the post-transplant or IV-ERT setting, are not well-established. Recently, Pal and colleagues compared post-intervention uGAG levels with the presence and severity of sleep-disordered breathing among treated MPS-IH patients^10^. Similar to our findings, they showed there were significant differences in uGAG among MPS-IH patients who demonstrated high versus low/no inhibitory ADAs. They further showed that high inhibitory antibody response correlated with greater sleep-disordered breathing^10^. Whether the magnitude of uGAG change correlates to other clinical disease manifestations (orthopedic, cardiac, neurologic, cognitive, etc.) is not yet clear, and the use of ERT following HCT has not been supported nor recommended.

Xue *et al*. showed there was in inverse relationship between the ADA response and percent reduction in urinary GAG in a meta-analysis of MPS I patients receiving ERT (patients had not undergone HCT)^14^. Unlike the clinical findings of Pal *et al*., no relationships between the ADA response and changes in percent predicted forced vital capacity and six-minute walk test were observed^14^.

We show the potential for IV-ERT augmentation to significantly reduce uGAG in post-transplant MPS-IH patients. Correlation of uGAG response with clinical endpoints for this cohort are important and are reported separately^16^. However, we also show that high inhibitory ADA response in this setting leads to increased uGAG. Both ADA and uGAG response beyond termination of IV-ERT at study’s end are not currently known. Despite a biochemical change in patients undergoing ERT post-transplant and because of the concern for ADA development, uGAG, ADA and inhibitory antibody responses should be monitored. Given the chronic nature of bony disease in MPS-IH, a longer-term study on the effect of ERT post-transplant should be performed before post-transplant ERT can be firmly recommended.

## Methods

### Participants

Patients with a diagnosis of Hurler syndrome and ≥ 2 years out from HCT were eligible for this study. This study was approved by the Committee on the Use of Human Subjects in Research at the University of Minnesota, and all experiments were performed in accordance with relevant guidelines and regulations by the Committees on the Use of Human Subjects in Research at the University of Minnesota. The methods were carried out in accordance with the relevant guidelines and regulations. Informed consent was obtained from all participants and/or their legal guardian/s. This study was registered on clinicaltrials.gov (NCT 01173016, July 30^th^, 2010). Eleven patients were enrolled. One patient dropped out at 6 months due to the time involved in ERT infusions and uncertainty about continuation in the study (their data is not included in our analyses). Ten participants completed the study. Following informed consent, patients underwent a baseline evaluation, and began IV-ERT (laronidase, 0.58 mg/kg IV weekly) for 104 consecutive weeks. Infusion-related toxicities were recorded. Demographic, transplant-related, and biochemical data including leukocyte α-L-iduronidase activity as measured by a clinical laboratory assay were recorded at baseline.

### Chimerism

Chimerism was assessed on the myeloid fraction (with ≥98% purity) of peripheral blood by our clinical laboratory after positive immune-magnetic selection of the CD15^+^ cell population. In cases where positive selection did not occur, whole blood samples were analyzed (ID 006). Donor chimerism was performed by polymerase chain reaction amplification of donor-specific short tandem nucleotide repeats.

### Anti-drug antibody

Patients underwent longitudinal evaluations of donor hematopoietic chimerism, laronidase ADA titers, and uGAG at baseline (prior to first laronidase dose) and every 3 months (ADA titers and uGAG) or every 6 months (chimerism) throughout the 2-year treatment duration. ADA titers were assessed in parallel by two methods. In the first, plasma samples were submitted to the drug manufacturer for the commercially-available (Com) determination of total ADA titer using end-point titration^14^. For the second method, we independently (Ind) evaluated concurrent plasma samples for ADA titer as previously described^10^. Furthermore, for all patients who mounted an ADA titer ≥1,000 at any time-point on study as measured by the independent assay, we determined ADA inhibitory capacity at two time-points: at ADA titer peak and at the end-of-study (month 24). As previously described, this inhibitory assay registered ADA’s sum total interference with both enzyme cellular uptake and catalytic function^10^. Patients were considered to have high ADA response if their maximum titer on study was ≥4,000 (using either ADA method) and the mean inhibition (averaged between the inhibition at the ADA titer peak and at month 24) was ≥30%, as previously described^10^. All other patients were considered to demonstrate low/non-significant ADA responses. Quantitative urinary GAG excretion was performed using the Sensi-Pro assay to measure non-reducing ends (NREs) characteristic for MPS-IH: I0S0, I0S6, and total HS concentration^17,18^.

### Statistical analysis

Patients with measurable laronidase ADA at study entry (n = 3) were compared to those without baseline ADA (n = 7) for the following pre-IV-ERT parameters: donor hematopoietic chimerism level and urinary excretion of HS, and the non-reducing ends I0S0, and I0S6. Differences in the mean values between the two groups were assessed with t-tests. Changes in donor hematopoietic chimerism and uGAG over time (HS, I0S0, and I0S6) were analyzed using a generalized estimating equations model (GEE), accounting for correlation among repeated measurements from the same subject. Outcome variables were log-transformed to stabilize residual variance, thus geometric means (GM) were calculated. Estimations of the change in uGAG at study’s end (month 24) compared to entry (baseline) were expressed as GM ratios (null hypothesis that the true GM ratio = 1, or that no change in uGAG would occur). Changes in donor hematopoietic chimerism between baseline and 24 months were analyzed in a similar manner^19^. Analyses were performed using SAS version 9.3 (SAS Institute, Cary, NC, USA).

## Data Availability

The datasets generated during and/or analyzed during the current study are available from the corresponding author on reasonable request.
